# Introduction to CATCH-IT Reports: Critically Appraised Topics in Communication, Health Informatics, and Technology

**DOI:** 10.2196/jmir.6.4.e49

**Published:** 2004-12-31

**Authors:** Gunther Eysenbach, Cameron Norman

**Affiliations:** ^1^Centre for Global eHealth InnovationUniversity Health NetworkToronto ONCanada

**Keywords:** Internet, information storage and retrieval, evidence-based medicine, periodicals, journal club, research methods

## Abstract

EHealth has developed largely from an interdisciplinary framework and, as such, does not have a “home” discipline. The absence of this home discipline has allowed eHealth research to be published widely in journals ranging from the medical sciences, to engineering, to social science or to business and policy studies. The result of this fragmented, decentralized literature base is that researchers are not always aware of important papers published in other areas and journals. With this issue the Journal of Medical Internet Research is inaugurating a new article category which we call “CATCH-IT Reports” (Critically Appraised Topics in Communication, Health Informatics, and Technology). We hope these reports will draw attention to important work published in other (sometimes obscure) journals, provide a platform for discussion around results and methodological issues in eHealth research, and help to develop a framework for evidence-based eHealth. CATCH-IT Reports arise from “journal club” - like sessions founded in February 2003 at the Centre for Global eHealth Innovation. We invite other research institutions to create similar journal clubs and to write up and submit to this journal critiques in the form of CATCH-IT Reports.

## Introduction: Information Scatter in eHealth Research

While health informatics is widely seen as a discipline with the potential for making health care more effective by advancing the introduction of medical evidence into clinical practice, information professionals are not always known for their optimal utilization of research findings in their own area of specialization. One of the barriers for keeping on top of research findings in health informatics is that even for information professionals trained in retrieving, organizing, and filtering information, it is difficult to keep pace with the scattered literature in this rapidly expanding field. With information technology having penetrated virtually every field of medicine, pertinent papers appear scattered in many different journals. in particular if we consider the broad definition of medical informatics as “the field that deals with the storage, retrieval, and optimal use of biomedical information, data, and knowledge for problem solving and decision making or in information delivery” [1], or the even broader definition of “eHealth”, which would also include for example papers dealing with the role of the Internet for information dissemination, data collection, and decision making for health professionals and consumers, and impact of the Internet on health behavior and well-being of people with a public health focus [2, 3]. In a search conducted on December 30, 2004 for journal articles published in 2003/2004 with “Internet” as a major MeSH keyword, we identified 1702 papers. (Note that at this time not all 2004 articles are yet Medline-indexed; thus this data contains only a subset of the papers published in 2004. We also excluded 70 articles published in *Internet Healthcare Strategies* as this journal does not publish original papers).

The 1702 papers were scattered across 685 different journals (Table 1 lists the top 20 journals publishing most of the papers). Reading the top two journals in this field (*Journal of Medical Internet Research *and *Cyberpsychology & Behavior: the Impact of the Internet, Multimedia and Virtual Reality on Behavior and Society*) as well as the two proceedings volumes from major medical informatics conferences (the proceedings of the American Medical Informatics Association fall conferences, and the conference proceedings of the European Federation of Medical Informatics *Studies in Health Technology and Informatics*) would keep readers informed of approximately 10% of the work published in this area.  One would have to read papers in 36 different journals to cover 33% of the articles, 92 different journals to cover 50%, 190 journals to cover 66%, and 344 journals to cover 80% of all articles  (Figure 1). While such a distribution, where a small group of core journals would provide 1/3 of the articles on that subject, a medium number of less-core journals would provide another 1/3 of the articles on that subject, and a large number peripheral journals would provide the final 1/3 of the articles on that subject, is typical (known as “Law of Scatter” or “Bradford's Distribution”), the extent of scatter in the field of eHealth is extreme, with a very long tail. While the *Journal of Medical Internet Research *ranks as the top journal which published more papers related to the Internet in medicine than any other journal, it still “only” publishes about 3% of the total number of papers in this field (which, by the way, we hope are the best 3%, and which is a proportion we hope to increase significantly over the next years).

**Table 1:** Distribution of articles published 2003/2004 and Medline-indexed with the keyword “Internet” as major MeSH (Medical Subject Heading) among the top 20 journals (a total of N=1702 papers were scattered in 685 different journals, not all shown here)

**Rank**	**Journal Title**	**Number of Articles Published 2003/2004 with “Internet” as Major MeSH Keyword**	**Percentage of Total Articles (N=1702)**	**Cumulative Percentage**
1	J Med Internet Res	47	2.76%	2.76%
2	AMIA Annu Symp Proc	44	2.59%	5.35%
3	Stud Health Technol Inform	39	2.29%	7.64%
4	Cyberpsychol Behav	39	2.29%	9.93%
5	Bioinformatics	31	1.82%	11.75%
6	Int J Med Inform	21	1.23%	12.98%
7	Health Manag Technol	20	1.18%	14.16%
8	J Telemed Telecare	19	1.12%	15.28%
9	Comput Inform Nurs	18	1.06%	16.33%
10	Health Data Manag	18	1.06%	17.39%
11	Health Info Libr J	16	0.94%	18.33%
12	Behav Res Methods Instrum Comput	16	0.94%	19.27%
13	BMJ	15	0.88%	20.15%
14	Med Ref Serv Q	15	0.88%	21.03%
15	Profiles Healthc Mark	13	0.76%	21.80%
16	J Med Syst	12	0.71%	22.50%
17	Nurse Educ	11	0.65%	23.15%
18	J Nurs Educ	11	0.65%	23.80%
19	Med Econ	11	0.65%	24.44%
20	Health Serv J	10	0.59%	25.03%

**Figure 1:** Information scatter in eHealth: Distribution of “Internet”-related papers published in 2003/2004 by journal (with some prominent journals highlighted). While the *Journal of Medical Internet Research *attracts more pertinent papers in this field than any other journal, the majority of the literature remains scattered (this figure does not even take into account non-Medline indexed journals e.g. from engineering or the social sciences).

It becomes clear that one has to monitor a large number and broad spectrum of journals in order to stay abreast of the most important developments in the evolving field of eHealth. EHealth is in a unique position in that it has developed largely from an interdisciplinary framework and, as such, does not have a “home” discipline (even “medical informatics” is only a part of the broader eHealth scene). The absence of this home discipline has allowed eHealth research to be published widely in journals ranging from the medical sciences to engineering, to social sciences and to business and policy studies. The aforementioned analysis does not even take into account articles published in journals which are not indexed in Medline. The result of this fragmented, decentralized literature base is that researchers are not always aware of important papers published in other disciplines and journals.

## eHealth Journal Club and CATCH-IT Reports

With this issue, the *Journal of Medical Internet Research* is inaugurating a new article category which we call “CATCH-IT Reports” (Critically Appraised Topics in Communication, Health Informatics, and Technology). With this new article series we hope to draw attention to important work published in other (sometimes obscure) journals, and provide a platform for discussion around methodological issues in eHealth research.

The reports arise from “journal club” - like CATCH-IT sessions founded in February 2003 at the Centre for Global eHealth Innovation by Gunther Eysenbach with the goal of bringing together researchers, students, faculty, researchers, and health professionals interested in furthering understanding of eHealth through the process of critically appraising and discussing current eHealth research. The objectives of these bi-weekly sessions and the CATCH-IT Reports are

- To train researchers, students, and faculty in critical appraisal skills and to encourage an evidence-based approach to the medical informatics literature
- To bring to the attention of the eHealth community important and timely issues and publications concerning evidence and issues in the field
- To provide, in a systematic fashion, critical analyses of publications and eHealth trials
- To identify pressing research issues and to stimulate thinking about methodological issues in eHealth.

The CATCH-IT review group at the Centre for Global eHealth Innovation comprises individuals who all have experience in developing, deploying and evaluating eHealth interventions. What we lack in terms of length of experience (as we all do in this new field) we make up for in breadth of experience. Our group consists of researchers and practitioners from many different disciplines, cultures, ages, and roles. We are scientists, practitioners, professors, and students. All of us are consumers. Some of us are new to the process of critical appraisal of research while others have been at the forefront of developing methods for evidence-based medicine since the term was first coined. In short, we represent a diverse cross section of eHealth consumers.

Each session is prepared by one of the participants taking on the role of facilitator. The facilitator selects a recent paper from the current body of literature (as a guideline it should not be older than 6 months, in exceptional cases up to 12 months) and circulates the paper to all participants at least 1 week before the CATCH-IT session.

Selection criteria for papers discussed in a CATCH-IT report include one or more of the following:

- High quality papers with great potential impact on one or more groups of decision makers in the health system *or*
- Papers illustrating methodological flaws worth discussing (seeking to prevent them in future studies)
- Papers providing an elegant solution to a (methodological) problem or otherwise addressing timely methodological issues or problems
- Illustration of new ideas or concepts that could represent food for reflection and discussion
- Direct impact on ongoing research (of the facilitator or elsewhere).

At the session, the facilitator first presents the paper, which is then critically discussed in the group under aspects such as validity and importance of the paper. Often, new research ideas are generated in the process, or methodological problems are unearthed. Almost always questions remain open as articles are often incompletely reported. We often ask ourselves the question, “How could the peer reviewer/editor miss this?”

Minutes of the discussions are kept, and the facilitator writes up a short report, typically 1000 to 1500 words in length. In the future, we will publish the best of these reports in this journal. The reports will be sent to the author of the original paper and he will be invited to respond to the report. A general template for CATCH-IT Reports will be developed during the project, but generally each published CATCH-IT Report will address the following questions:

- Why is the paper interesting, why was it picked?
- For whom is this paper interesting, and why?
- What were the intervention, setting, outcome measures, results, and conclusions of the authors?
- Background information – what's not written in the paper is…
- What are the methodological issues, and is the result valid?
- What can health professionals learn from this study?
- What can consumers learn from this study?
- What can policy makers learn from this study?
- What can researchers learn from this study?
- What further research is required?
- What questions for the author arise?

One of our goals is to produce reports based on our discussions and reviews in order to promote better research and to foster further debate and discussion on ways to create the best evidence for the use of eHealth. The focus of the first report, published in this issue [4], is on the article's contribution to our understanding of eHealth - it's efficacy, effectiveness or potential use in research. We also hope that from this work we will be able to compile some guidelines for specific eHealth methodologies and approaches to development and evaluation of eHealth innovations. This is not to suggest that guidelines for evaluating eHealth research do not exist. The CONSORT statement [5], the guidelines for interactive health communication from SciPICH3 [6], and many discipline-specific research guidelines all offer us some clues on what to look for when evaluating these articles. It is our hope that, over time, we can further refine such guidelines to meet the changing climate of eHealth, and perhaps work on specific guidelines for particular problems, such as the CHERRIES statement for Web-based survey research [7].

While forthcoming CATCH-IT papers will be primarily produced by graduate students and faculty at the Centre for Global eHealth Innovation, we invite other research institutions to create similar journal clubs and to write up and submit critiques and discussion pieces of the current eHealth literature in the form of CATCH-IT Reports.
